# Type I gamma phosphatidylinositol phosphate kinase i5 suppresses YAP1 signaling

**DOI:** 10.1016/j.jbc.2025.110573

**Published:** 2025-08-08

**Authors:** Chinmoy Ghosh, Ruchi Kakar, Matthew Bavuso, Huizhi Wang, Yue Sun

**Affiliations:** 1 Department of Oral and Craniofacial Molecular Biology, Philips Institute for Oral Health Research, School of Dentistry, Virginia Commonwealth University, Richmond, Virginia, USA; 2 Massey Cancer Center, Virginia Commonwealth University, Richmond, Virginia, USA

**Keywords:** 14-3-3, PIPKIγi5, PI4,5P_2_, YAP1, tumorsphere

## Abstract

Dysregulation of the Hippo/Yes-associated protein (YAP) signaling pathway has been associated with several diseases, including cancer, neurological disorders, and cardiovascular conditions. However, the precise molecular mechanisms governing Hippo/YAP signaling are not fully understood, and additional regulators of this pathway need to be identified. Our research has identified type I gamma phosphatidylinositol phosphate kinase i5 (PIPKIγi5) as a regulator of Hippo/YAP signaling. PIPKIγi5 is a kinase responsible for synthesizing phosphatidylinositol-4,5-bisphosphate (PI4,5P_2_). By directly interacting with YAP1, PIPKIγi5 prevents the nuclear translocation of YAP1, thereby suppressing YAP1-mediated gene transcription. Thus, PIPKIγi5 functions as a suppressor of YAP1-mediated signaling. The kinase activity of PIPKIγi5, which generates PI4,5P_2_, is essential for controlling YAP1 function. PI4,5P_2_ promotes the interaction of YAP1 with the 14-3-3 protein, which retains YAP1 in the cytosol. Given the role of YAP1 signaling in cancer cell stemness, depletion of PIPKIγi5 enhances YAP1 signaling and promotes tumorsphere formation in head and neck squamous cell carcinoma. These findings highlight a PI4,5P_2_-modulated signaling nexus that exerts specific control over Hippo/YAP signaling and its biological functions.

The Yes-associated protein 1 (YAP1) is a transcriptional coactivator and a key component of the Hippo/YAP pathway (1, 2). This pathway is essential for maintaining normal tissue development by regulating cell contact–mediated inhibitory signaling (3, 4). Upon activation, YAP1 translocates from the cytosol to the nucleus, where it associates with transcriptional enhanced associate domain (TEAD) family transcription factors to mediate the expression of target genes (5, 6, 7). Through this mechanism, YAP1 signaling governs processes, such as cell proliferation, differentiation, and organ regeneration (4). The activation of YAP1 must be tightly controlled, as its dysregulation is implicated in various diseases, including cancer, cardiovascular diseases, neurological disorders, and immune dysfunction (8, 9, 10, 11). Notably, YAP1 is hyperactivated in many cancers, where it sustains stem cell properties and enhances cancer stem cell traits, contributing to therapeutic resistance, recurrence, and metastasis (12, 13, 14). As a result, YAP1-targeting strategies have emerged as a focus for anticancer therapies. Current approaches primarily aim to disrupt the YAP1–TEAD interaction (15). However, the clinical success of these inhibitors has been limited (16). This highlights an unmet need for a deeper understanding of the regulatory mechanisms governing YAP1 activity and the identification of novel regulators as potential drug targets.

Phosphatidylinositol-4,5-bisphosphate (PI4,5P_2_) is a vital component of membrane phospholipids (17). It serves not only as a precursor for producing key signaling molecules, such as phosphatidylinositol-3,4,5-trisphosphate, inositol-1,4,5-trisphosphate, and diacylglycerol but also functions as a critical messenger itself (18). By interacting with effector proteins and modulating their biological activities, PI4,5P_2_ regulates a wide range of cellular processes, including vesicular trafficking, cytoskeletal rearrangement, ion channel regulation, cell growth, and protein metabolism (19). Recent evidence suggests that YAP1 is a PI4,5P_2_ effector protein (20). Phosphatidylinositol 4-phosphate 5-kinase type 1α (PIPKIα), a kinase responsible for generating PI4,5P_2_, plays a critical role in this context. In the nucleus, PIPKIα interacts with YAP1, and the PI4,5P_2_ it produces facilitates the interaction of YAP1 with TEAD transcription factors, thereby enhancing YAP1–TEAD-mediated gene transcription (20). This indicates that nuclear PI4,5P_2_ can promote the activation of YAP1 signaling.

PIPKIα belongs to the type I phosphoinositide phosphate kinase (PIPKI) family, which catalyzes the phosphorylation of phosphatidylinositol 4-phosphate to generate PI4,5P_2_ (18, 21). In addition to PIPKIα, the PIPKI family includes two other isoforms, PIPKIβ and PIPKIγ (22). These isoforms exhibit distinct subcellular localizations (21, 23). Interestingly, PIPKIα- or PIPKIβ-knockout mice are viable and can survive into adulthood (24, 25, 26). In contrast, pan-knockout mice for PIPKIγ experience perinatal lethality (27), suggesting that PIPKIγ has unique biological functions that cannot be compensated by other PIPKI isoforms, despite all isoforms contributing to PI4,5P_2_ production. The PIPKIγ gene (PIP5K1C) undergoes alternative splicing, resulting in multiple splice variants. At least six variants named PIPKIγi1 through PIPKIγi6 are expressed in humans (28, 29). These variants share the same N-terminal region and kinase domain but differ in their far C-terminal extensions. The distinct C-terminal regions allow these PIPKIγ variants to interact with specific effector proteins, enabling their recruitment to particular subcellular locations where they perform diverse biological functions (30, 31, 32, 33, 34).

Intriguingly, our current research demonstrates that PIPKIγi5 (type I gamma phosphatidylinositol phosphate 5-kinase i5), a splice variant of PIPKIγ, plays a distinct role in modulating YAP1 signaling compared with PIPKIα. While PIPKIα promotes YAP1 signaling in the nucleus, PIPKIγi5 modulates YAP1 in the cytosol. The production of PI4,5P_2_ by PIPKIγi5 enhances the interaction between YAP1 and 14-3-3, effectively sequestering YAP1 in the cytosol. Consequently, PIPKIγi5 inhibits YAP1 nuclear translocation and suppresses YAP1-mediated gene transcription. These findings highlight that different PI4,5P_2_-producing kinases can modulate YAP1 signaling in a subcellular location–dependent manner.

## Results

### YAP1 specifically interacts with PIPKIγi5

**Figure 1:**
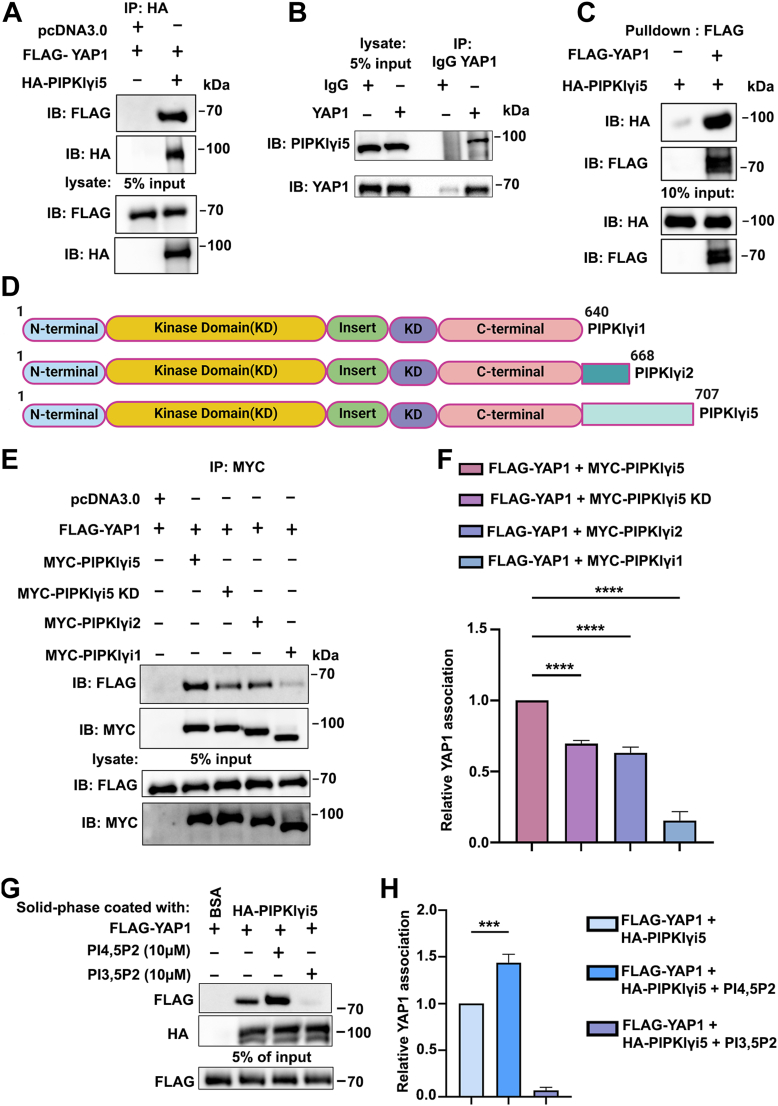
**PIPKIγi5 interacts with YAP1.***A*, HEK-293 cells coexpressing HA-PIPKIγi5 and FLAG-YAP1 were subjected to HA antibody immunoprecipitation and subsequently immunoblotted with the indicated antibodies. *B*, CAL27 cells were immunoprecipitated utilizing YAP1 antibody, then followed by immunoblotting with the indicated antibodies. *C*, recombinant FLAG-YAP1 and HA-PIPKIγi5 proteins (purified *via* Halo-tag system) were subjected into *in vitro* pull-down assays with Anti-FLAG Magnetic Beads. *D*, the diagram depicts the domain architecture of PIPKIγi1, i2, and i5. Developed with BioRender.com. *E*, the Myc-tagged PIPKIγi1, PIPKIγi2, PIPKIγi5, or PIPKIγi5 kinase dead mutants (D316A; referred to as PIPKIγi5KD) were coexpressed with FLAG-YAP1 in HEK-293 cells, followed by immunoprecipitation from cell lysates using anti-Myc antibody. *F*, quantification of YAP1 interaction with PIPKIγi1, PIPKIγi2, PIPKIγi5, or PIPKIγi5KD. *G*, the interaction between purified HA-PIPKIγi5 and FLAG-YAP1 was assessed using an *in vitro* solid-phase binding assay, with or without the presence of PI3,5P_2_ or PI4,5P_2_ as indicated. *H*, quantification of the interaction between PIPKIγi5 and YAP1 in the solid-phase binding assay. The values presented in the graphs indicate the mean ± SD from three independent experiments. Statistical significance was determined using one-way ANOVA and Tukey’s HSD (*F* and *H*) (∗∗∗*p* < 0.0005; ∗∗∗∗*p* < 0.0001). HEK-293, human embryonic kidney 293 cell line; HSD, honestly significant difference test; PIPKIγi5, type I gamma phosphatidylinositol phosphate kinase i5; YAP1, Yes-associated protein 1.

During the investigation of potential PIPKIγi5 effector proteins, we identified an interaction between YAP1 and PIPKIγi5. As shown in Figure 1*A*, FLAG-tagged YAP1 was coexpressed with HA-tagged PIPKIγi5 in human embryonic kidney 293 (HEK-293) cells, and a coimmunoprecipitation (IP) assay demonstrated that FLAG-YAP1 could be co-IPed with HA-PIPKIγi5. This result indicates that PIPKIγi5 interacts with YAP1. To further validate the PIPKIγi5–YAP1 interaction, endogenous YAP1 was immunoprecipitated from CAL27 cell lysates, and endogenous PIPKIγi5 was detected within the YAP1 complex *via* Western blot analysis (Fig. 1*B*). In addition, to determine whether PIPKIγi5 directly binds to YAP1, purified FLAG-YAP1 and HA-PIPKIγi5 recombinant proteins (Fig. S1) were used in the *in vitro* binding assay. As shown in Figure 1*C*, purified HA-PIPKIγi5 was pulled down by purified FLAG-YAP1, confirming a direct interaction between PIPKIγi5 and YAP1.

To determine the specificity of the PIPKIγi5–YAP1 interaction, we compared the ability of other PIPKIγ splice variants, such as PIPKIγi1 and PIPKIγi2, to interact with YAP1. As illustrated in Figure 1*D*, these PIPKIγ splice variants differ in the number of amino acids: PIPKIγi1 has 640 amino acids, PIPKIγi2 has 668 amino acids, and PIPKIγi5 has 707 amino acids. Notably, all three variants share an identical sequence for the first 640 amino acids, whereas PIPKIγi2 and PIPKIγi5 have distinct C-terminal extensions built upon the core structure of PIPKIγi1 (Fig. 1*D*). Our results demonstrate that PIPKIγi1 does not bind to YAP1, and the interaction of PIPKIγi2 with YAP1 is significantly weaker compared with PIPKIγi5 (Fig. 1, *E* and *F*). These findings confirm the specificity of the PIPKIγi5–YAP1 interaction and indicate that the unique C-terminal region of PIPKIγi5 is critical for its ability to bind YAP1. In addition, a kinase-dead mutant of PIPKIγi5 (D316A; referred to as PIPKIγi5KD) showed a marked deficiency in binding YAP1 (Fig. 1, *E* and *F*). YAP1 is a PI4,5P_2_ effector protein capable of binding PI4,5P_2_, which facilitates its interaction with the effectors such as TEAD family transcription factors (20). It is possible that the kinase activity of PIPKIγi5, which is responsible for generating PI4,5P_2_, could regulate the PIPKIγi5 interaction with YAP1. To test this, a solid-phase *in vitro* binding assay was performed using purified recombinant FLAG-YAP1 and HA-PIPKIγi5 proteins to examine the effects of PI4,5P_2_ on YAP1–PIPKIγi5 interaction. As shown in Figure 1, *G* and *H*, the addition of PI4,5P_2_ significantly enhanced the interaction between YAP1 and PIPKIγi5. As a control, another phosphoinositide, phosphatidylinositol-3,5-bisphosphate (PI3,5P_2_), was tested and found unable to enhance the interaction between YAP1 and PIPKIγi5, demonstrating the specificity of PI4,5P_2_ in modulating the YAP1–PIPKIγi5 interaction (Fig. 1, *G* and *H*).

### Characterization of the binding regions responsible for PIPKIγi5–YAP1 interaction

**Figure 2:**
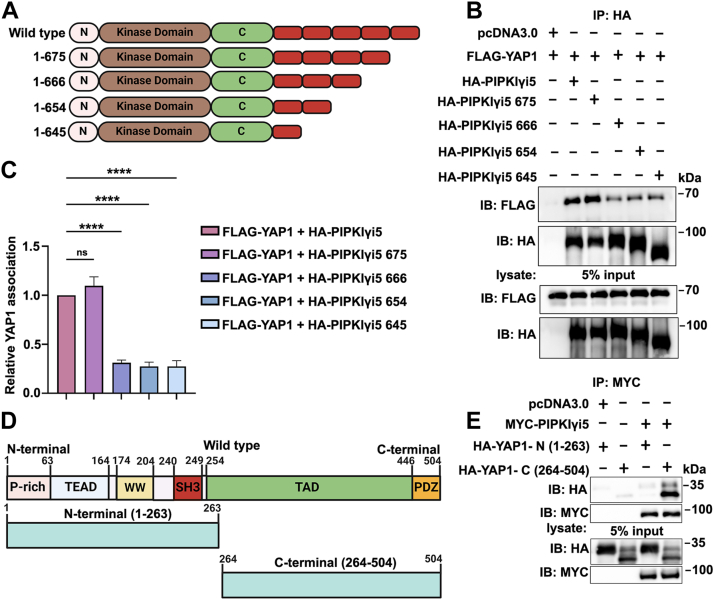
**Identification of the binding regions responsible for the interaction between PIPKIγi5 and YAP1.***A*, a diagrammatic representation of the sequence of PIPKIγi5 C-terminal truncation mutants. Schematic diagram was created using BioRender.com. *B*, FLAG-YAP1 was coexpressed with HA-tagged wildtype PIPKIγi5 or a variety of PIPKIγi5 C-terminal truncation mutants, HA antibody was used for immunoprecipitation from cell lysates. *C*, levels of YAP1 interaction with wildtype PIPKIγi5 or PIPKIγi5 C-terminal truncation mutants were quantified. *D*, schematic representation of the domains of YAP1 and its truncation mutants. Schematic diagram was created using BioRender.com. *E*, MYC-PIPKIγi5 was coexpressed with HA-YAP1-N (1-263) or HA-YAP1-C (264-504), and MYC antibody was used for immunoprecipitation from cell lysates. The values presented in the graphs indicate the mean ± SD derived from three independent experiments. One-way ANOVA and Tukey’s HSD (*C*) (∗∗∗∗*p* < 0.0001, and ns, nonsignificant). C, C terminus; HSD, honestly significant difference test; KD, kinase domain; N, N terminus; PDZ, PDZ-binding motif; PIPKIγi5, type I gamma phosphatidylinositol phosphate kinase i5; P-rich, proline-rich; SH3, SH3 domain; TAD, transcription activation domain; TEAD, transcriptional enhanced associate domain; WW, tryptophan–tryptophan domain; YAP1, Yes-associated protein 1.

To further identify the regions within the PIPKIγi5-specific C terminus responsible for binding to YAP1, a series of truncation mutants were generated (Fig. 2*A*), and their ability to interact with YAP1 was assessed by co-IP experiments (Fig. 2, *B* and *C*). The truncation mutant PIPKIγi5_675, in which the C terminus beyond amino acid 675 was deleted, retained full binding ability to YAP1, comparable to the wildtype PIPKIγi5. However, the interaction of the truncation mutant PIPKIγi5_666, in which the C terminus beyond amino acid 666 was deleted, was significantly reduced (Fig. 2, *B* and *C*). These findings suggest that the amino acid sequence spanning residues 666 to 675 in the PIPKIγi5 C terminus is critical for its interaction with YAP1.

To identify the region of YAP1 responsible for interacting with PIPKIγi5, truncation mutants of YAP1 were generated, encompassing either the N-terminal portion (YAP1-N, residues 1–263) or the C-terminal portion (YAP1-C, residues 264–504) (Fig. 2*D*). YAP1-N includes the proline-rich region, TEAD-binding domain, WW domain, and SH3-binding motif, whereas YAP1-C contains the transcriptional activation domain and PDZ-binding motif. Co-IP experiments revealed that only YAP1-C was capable of binding to PIPKIγi5 (Fig. 2*E*), indicating that the C-terminal region of YAP1 mediates its interaction with PIPKIγi5.

### Depletion of PIPKIγi5 enhances the YAP1 target gene expression

**Figure 3:**
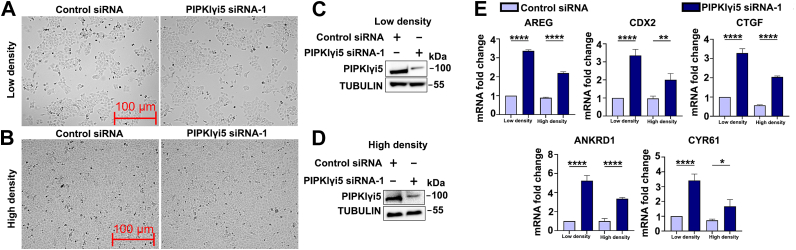
**Loss of PIPKIγi5 enhances YAP1 target gene expression.** Control siRNA or PIPKIγi5 siRNA-1 were transfected into CAL27 cells in low cell density (*e.g.*, sparsity) (*A*) or high cell density (*e.g.*, confluence) (*B*) conditions. The PIPKIγi5 protein levels were examined by Western blot in sparsity (*C*) and confluence cells (*D*). The mRNA levels of indicated YAP1 target genes were examined by real-time PCR (*E*). The values shown on graphs represent the mean ± SD from three independent experiments. One-way ANOVA and Tukey’s HSD (*E*) (∗*p* < 0.05; ∗∗*p* < 0.001; and ∗∗∗∗*p* < 0.0001). HSD, honestly significant difference test; PIPKIγi5, type I gamma phosphatidylinositol phosphate kinase i5; YAP1, Yes-associated protein 1.

As a transcriptional coactivator, YAP1 regulates the transcription of many genes involved in growth and survival (35). Since PIPKIγi5 directly interacts with YAP1, the effects of PIPKIγi5 knockdown on YAP1 target gene expression were examined. Scramble control siRNA or PIPKIγi5-specific siRNA (PIPKIγi5 siRNA-1) were transfected into the head and neck squamous cell carcinoma cell line CAL27. As YAP1 activation is sensitive to cell density, the effects of PIPKIγi5-knockdown were tested in both low cell density (*e.g.*, sparsity) and high cell density (*e.g.*, confluence) conditions (Fig. 3). As shown in Figure 3*E*, depletion of PIPKIγi5 in both sparsity and confluence conditions significantly increased the mRNA expression levels of YAP1 target genes, including AREG, CDX2, CTGF, ANKRD1, and CYR61. The effects of PIPKIγi5-knockdown on promoting these gene expression in sparsity condition is more robust than in confluence condition, which is consistent with that the confluence condition can inhibit YAP1 activation (Fig. 3*E*). The sparsity condition was then used for the rest of experiments examining the function of PIPKIγi5 in YAP1 signaling. The effects of PIPKIγi5 on the expression of these genes were dependent on YAP1, as PIPKIγi5-knockdown failed to enhance these gene expressions in YAP1-depleted CAL27 cells (Fig. S2). To exclude the possible siRNA off-target effects, another sequence of PIPKIγi5 siRNA (PIPKIγi5 siRNA-2) was used. PIPKIγi5 siRNA-2 could similarly knock down PIPKIγi5 expression and promote YAP1 target gene expression (Fig. S3).

Consistent with these mRNA changes, the protein expression levels of YAP1 target genes, such as CTGF and CYR61, were also significantly elevated in PIPKIγi5-knockdown CAL27 cells (Fig. S4, *A* and *B*), further supporting the role of PIPKIγi5 in regulating YAP1 target gene expression. In contrast, knockdown of PIPKIγi2 did not significantly alter the mRNA or protein levels of YAP1 target genes (Fig. S4, *A*–*C*), consistent with the weaker interaction between PIPKIγi2 and YAP1 compared with PIPKIγi5. Similarly, knockdown of PIPKIγi5, but not PIPKIγi2, in another head and neck squamous cell carcinoma cell line, UM-SCC-1, also significantly increased both the mRNA and protein levels of YAP1 target genes (Fig. S4, *D*–*F*). These results suggest that the role of PIPKIγi5 in modulating YAP1 target gene expression is not cell line specific.

### PIPKIγi5 facilitates YAP1 interaction with 14-3-3

**Figure 4:**
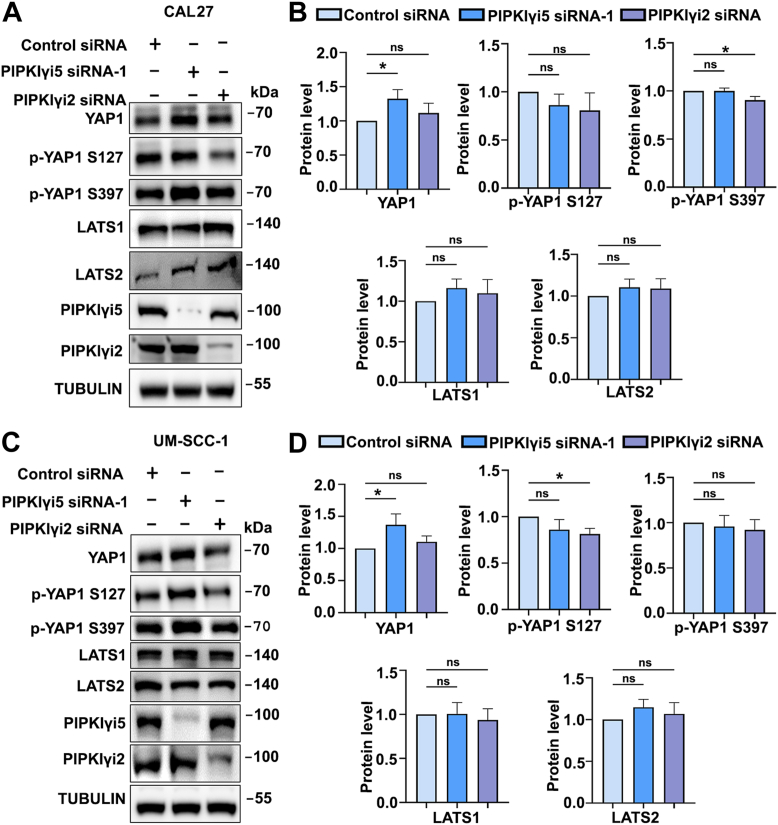
**Effects of PIPKIγi5 on YAP1 expression and phosphorylation.** CAL27 cells were transfected with either control, PIPKIγi5 siRNA-1, or PIPKIγi2 siRNA, and the specified protein levels were analyzed using Western blot (*A*). Quantification of total YAP1, YAP1 phosphorylation (S127 and S397), LATS1 and LATS2 in CAL27 cells (*B*). UM-SCC-1 cells were transfected with either control, PIPKIγi5 siRNA-1, or PIPKIγi2 siRNA, and the specified protein levels were analyzed using Western blot (*C*). Quantification of total YAP1, YAP1 phosphorylation (S127 and S397), LATS1 and LATS2 in UM-SCC-1 cells (*D*). The values shown on graphs represent the mean ± SD from three independent experiments. One-way ANOVA and Tukey’s HSD (*B*, *D*) (∗*p* < 0.05, and ns, nonsignificant). HSD, honestly significant difference test; LATS1/2, large tumor suppressor kinase 1 and 2; PIPKIγi5, type I gamma phosphatidylinositol phosphate kinase i5; YAP1, Yes-associated protein 1.

As a key downstream effector of the Hippo signaling pathway, YAP1 activation is regulated by the Hippo core kinase cascade (36). Large tumor suppressor kinases 1 and 2 (LATS1/2) suppress YAP1 activation by phosphorylating YAP1 at conserved serine residues (*e.g.*, S127 and S397). This phosphorylation facilitates the interaction of YAP1 with 14-3-3 proteins, sequestering YAP1 in the cytoplasm (37). In addition, LATS1/2-mediated YAP1 phosphorylation primes further phosphorylation by other kinases, ultimately leading to YAP1 polyubiquitination and degradation (38). Inhibition of LATS1/2 decreases YAP1 phosphorylation, thereby enhancing YAP1 signaling. The impact of PIPKIγi5 depletion on LATS1/2 expression and YAP1 phosphorylation was investigated. Knockdown of PIPKIγi5 by PIPKIγi5 siRNA-1 did not significantly affect the expression levels of LATS1 or LATS2 in either CAL27 (Fig. 4, *A* and *B*) or UM-SCC-1 cells (Fig. 4, *C* and *D*). PIPKIγi5-knockdown only slightly increased total YAP1 protein levels (Fig. 4, *A*–*D*). Using of another PIPKIγi5 siRNA (PIPKIγi5 siRNA-2) showed similar effects as PIPKIγi5 siRNA-1 (Fig. S5). These results suggest that PIPKIγi5 does not control YAP1 by modulating LATS1/2 expression or activation.

**Figure 5:**
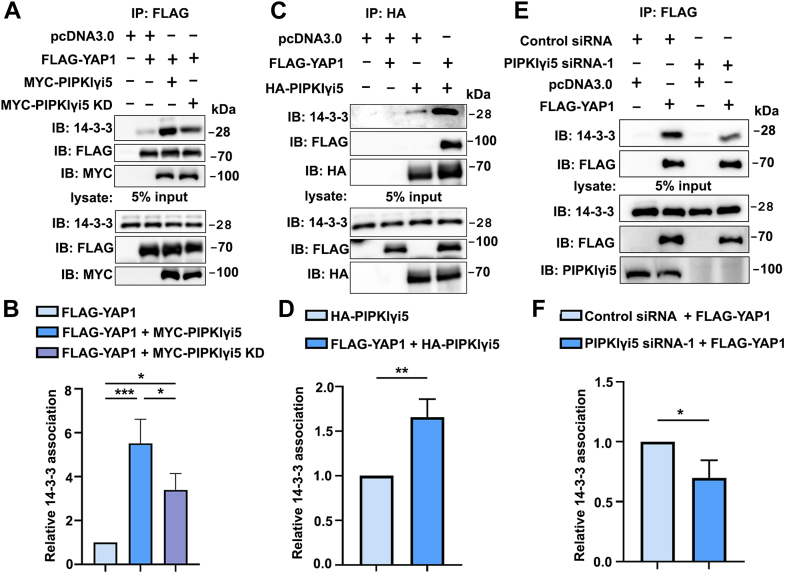
**PIPKIγi5 promotes YAP1 interaction with 14-3-3.***A*, FLAG-YAP1 were coexpressed with Myc-PIPKIγi5 or Myc-PIPKIγi5 KD in HEK-293 cells, and the cells were subjected to FLAG antibody immunoprecipitation and subsequently immunoblotted with the specified antibodies. *B*, quantification of YAP1 interaction with 14-3-3. *C*, HA-PIPKIγi5 was coexpressed with or without FLAG-YAP1 in HEK-293 cells, and then cells were subjected to HA antibody immunoprecipitation and subsequently immunoblotted with the specified antibodies. *D*, quantification of PIPKIγi5 interaction with 14-3-3. *E*, FLAG-YAP1 was expressed in control or PIPKIγi5-knockdown CAL27 cells and subjected to FLAG antibody immunoprecipitation and subsequently immunoblotted with the specified antibodies. *F*, quantification of YAP1 interaction with 14-3-3 in control or PIPKIγi5-knockdown cells. The values shown on graphs represent the mean ± SD from three independent experiments. One-way ANOVA and Tukey’s HSD (*B*) (∗*p* < 0.05; ∗∗∗*p* < 0.0005). Unpaired two-tailed Student’s *t* test (*D*, *F*) (∗*p* < 0.05; ∗∗*p* < 0.001). HEK-293, human embryonic kidney 293 cell line; HSD, honestly significant difference test; KD, kinase domain; PIPKIγi5, type I gamma phosphatidylinositol phosphate kinase i5; YAP1, Yes-associated protein 1.

The effects of PIPKIγi5 expression on YAP1 interaction with 14-3-3 protein were analyzed using a co-IP assay. FLAG-YAP1 was coexpressed with or without MYC-PIPKIγi5 or the kinase-dead mutant MYC-PIPKIγi5KD, and the interaction between YAP1 and endogenous 14-3-3 was assessed. As shown in Figure 5, *A* and *B*, PIPKIγi5 expression significantly enhanced YAP1 binding to 14-3-3. Expression of the PIPKIγi5KD mutant moderately increased the YAP1–14-3-3 interaction, but this effect was substantially weaker compared with wildtype PIPKIγi5. These findings suggest that PIPKIγi5 promotes YAP1 interaction with 14-3-3, and that its kinase activity, which generates PI4,5P_2_, contributes to this effect. Co-IP experiments further revealed that PIPKIγi5 can also interact with 14-3-3, and this interaction was further enhanced by the expression of YAP1 (Fig. 5, *C* and *D*). These results indicate that PIPKIγi5, YAP1, and 14-3-3 can form a complex. Moreover, depletion of PIPKIγi5 significantly reduced the interaction between YAP1 and 14-3-3 (Fig. 5, *E* and *F*), providing additional evidence for the role of PIPKIγi5 in regulating YAP1 binding to 14-3-3.

### PIPKIγi5 blocks YAP1 nuclear translocation

**Figure 6:**
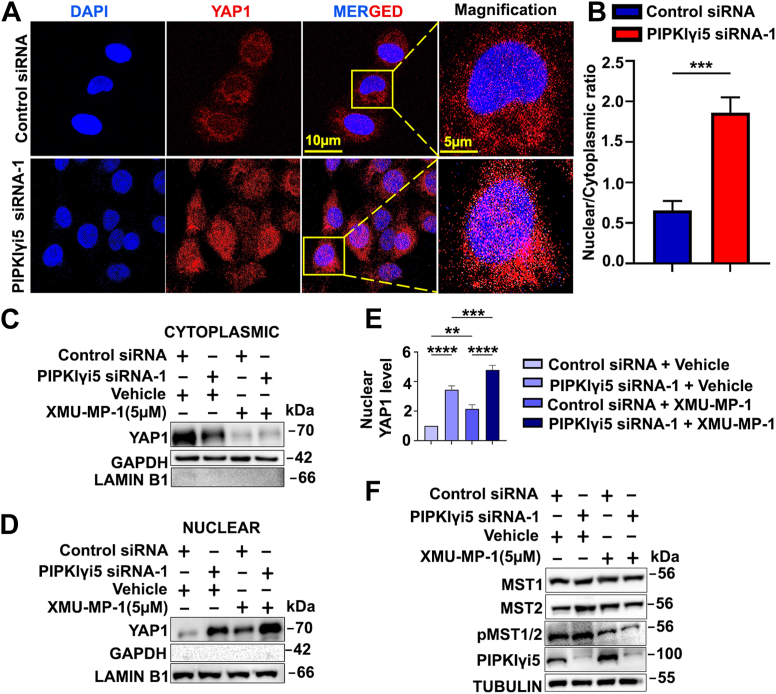
**Depletion of PIPKIγi5 increases the nuclear translocation of YAP1.** CAL27 cells were transfected with either control siRNA or PIPKIγi5 siRNA-1. *A*, control or PIPKIγi5-knockdown CAL27 cells were stained with YAP1 antibody. Nuclei were stained with DAPI (scale bars represent 10 μm). High magnifications of the respective framed regions were shown on the *right* (scale bars represent 5 μm). *B*, quantification of YAP1 nuclear IF staining. Error bars indicate mean ± SD. (n = 60 cells from three independent experiments). Control or PIPKIγi5-knockdown CAL27 cells were treated with or without the MST1/2 inhibitor XMU-MP-1, and then the cytoplasmic and nuclear fractions were isolated using nuclear extraction kits. Cytoplasmic constituents were subjected to immunoblotting using indicated antibodies (*C*). Nuclear components were subjected to immunoblotting using specified antibodies (*D*). The levels of nuclear YAP1 in control or PIPKIγi5-knockdown CAL27 cells were quantified (*E*). The PIPKIγi5 expression levels, MST1/2 expression levels, and MST1/2 phosphorylation (MST1 (Thr183)/MST2 (Thr180)) levels were examined by Western blot (*F*). The values shown on graphs represent the mean ± SD from three independent experiments. Unpaired two-tailed Student’s *t* test (*B*) (∗∗∗*p* < 0.0005). One-way ANOVA and Tukey’s HSD (*E*) (∗∗*p* < 0.001; ∗∗∗*p* < 0.0005, ∗∗∗∗*p* < 0.0001). DAPI, 4′,6-diamidino-2-phenylindole; HSD, honestly significant difference test; IF, immunofluorescence; MST1/2, mammalian STE20-like protein kinase 1/2; PIPKIγi5, type I gamma phosphatidylinositol phosphate kinase i5; YAP1, Yes-associated protein 1

The interaction between YAP1 and 14-3-3 retains YAP1 in the cytosol, preventing its nuclear translocation and thereby blocking the expression of YAP1 target genes (39). Since PIPKIγi5 promotes YAP1 interaction with 14-3-3, the effect of PIPKIγi5 expression on YAP1 nuclear translocation was investigated. At first, an immunofluorescence assay was performed to examine whether PIPKIγi5 affects YAP1 subcellular localization. As shown in Figure 6, *A* and *B*, knockdown of PIPKIγi5 significantly increased YAP1 distribution in the nucleus. These findings support the role of PIPKIγi5 in regulating YAP1 nuclear translocation.

The effects of PIPKIγi5-knockdown on YAP1 nuclear translocation were further validated by nuclear/cytosolic fractionation. Knockdown of PIPKIγi5 by PIPKIγi5 siRNA-1 markedly increased YAP1 nuclear translocation in rest status (Fig. 6, *C*–*E*), consistent with the observed reduction in YAP1 binding to 14-3-3 (Fig. 5, *E* and *F*) and the upregulation of YAP1 target gene expression (Fig. 3). Using of another PIPKIγi5 siRNA (PIPKIγi5 siRNA-2) had similar effect as PIPKIγi5 siRNA-1 in promoting YAP1 nuclear translocation (Fig. S6).

YAP1 activation and nuclear translocation can be stimulated by inhibiting the Hippo upstream kinases such as mammalian STE20-like protein kinase 1/2 (MST1/2) or LATS1/2. Treatment with MST1/2 inhibitor, XMU-XP-1, blocked MST1/2 activation as indicated by loss of MST1/2 phosphorylation (Fig. 6*F*). The inhibition of MST1/2 could enhance the YAP1 nuclear translocation in control cells (Fig. 6, *C*–*E*). In PIPKIγi5-knockdown cells, MST1/2 inhibition induced more robust YAP1 nuclear translocation compared with in control cells (Fig. 6, *C*–*E*). These results indicate that PIPKIγi5 can regulate Hippo kinase inhibition–induced YAP1 nuclear translocation.

### PI4,5P_2_ promotes YAP1 interaction with 14-3-3

**Figure 7:**
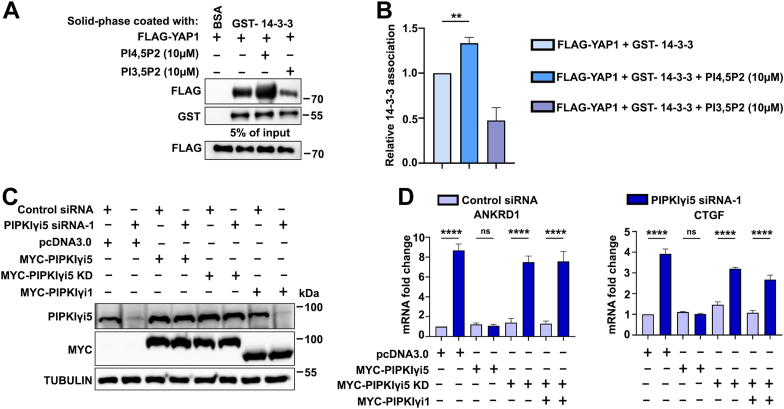
**The kinase activity of PIPKIγi5 is required for the regulation of YAP1.***A*, the interaction between purified GST-14-3-3 and FLAG-YAP1 was assessed using an *in vitro* solid-phase binding assay, with or without the presence of PI3,5P_2_ or PI4,5P_2_ as specified. *B*, quantification of the interaction between 14-3-3 and YAP1 in the solid-phase binding assay. Myc-PIPKIγi5, Myc-PIPKIγi5KD, or Myc-PIPKIγi1 were individually expressed in either control or PIPKIγi5-knockdown CAL27 cells, and then PIPKIγi5 expression was identified using Western blot analysis (*C*), the expression of YAP1 target genes, ANKRD1 and CTGF, was assessed using real-time PCR (*D*). The values shown on graphs represent the mean ± SD from three independent experiments. One-way ANOVA and Tukey’s HSD (*B*, *D*) (∗∗*p* < 0.001; ∗∗∗∗*p* < 0.0001; and ns, nonsignificant). HSD, honestly significant difference test; PI3,5P_2,_ phosphatidylinositol-3,5-bisphosphate; PI4,5P_2_, phosphatidylinositol-4,5-bisphosphate; PIPKIγi5, type I gamma phosphatidylinositol phosphate kinase i5; YAP1, Yes-associated protein 1.

To investigate whether PI4,5P_2_ modulates the interaction between YAP1 and 14-3-3, a solid-phase *in vitro* binding assay was performed using purified recombinant YAP1 and 14-3-3 proteins. As shown in Figure 7, *A* and *B*, the addition of PI4,5P_2_ significantly enhanced the interaction between YAP1 and 14-3-3. This finding underscores the importance of the kinase activity of PIPKIγi5 in generating PI4,5P_2_ to regulate YAP1–14-3-3 binding. This result aligns with the observation that wildtype PIPKIγi5 promotes YAP1 binding with 14-3-3 more effectively than the PIPKIγi5KD mutant (Fig. 5, *A* and *B*). As a control, another phosphoinositide, PI3,5P_2_, was tested and found unable to enhance the interaction between YAP1 and 14-3-3, demonstrating the specificity of PI4,5P_2_ in modulating the YAP1–14-3-3 interaction (Fig. 7, *A* and *B*).

To further confirm the effects of PIPKIγi5 kinase activity on YAP1 signaling, a knockdown and rescue experiment was conducted. Endogenous PIPKIγi5 expression was silenced using siRNA, followed by the expression of wildtype PIPKIγi5, the PIPKIγi5KD mutant, or PIPKIγi1 in PIPKIγi5-knockdown cells. PIPKIγi1 is a PIPKIγ splicing variant that cannot bind YAP1 (Fig. 1, *E* and *F*). As shown in Figure 7, *C* and *D*, the expression of wildtype PIPKIγi5 rescued the effects of PIPKIγi5 knockdown by suppressing the mRNA expression of YAP1 target genes, ANKRD1 and CTGF. In contrast, the expression of the PIPKIγi5KD mutant or PIPKIγi1 failed to suppress the mRNA levels of ANKRD1 and CTGF. These results underscore the requirement of PIPKIγi5 kinase activity and the ability to bind YAP1 in suppressing YAP1 signaling.

### Loss of PIPKIγi5 promotes tumorsphere formation

**Figure 8:**
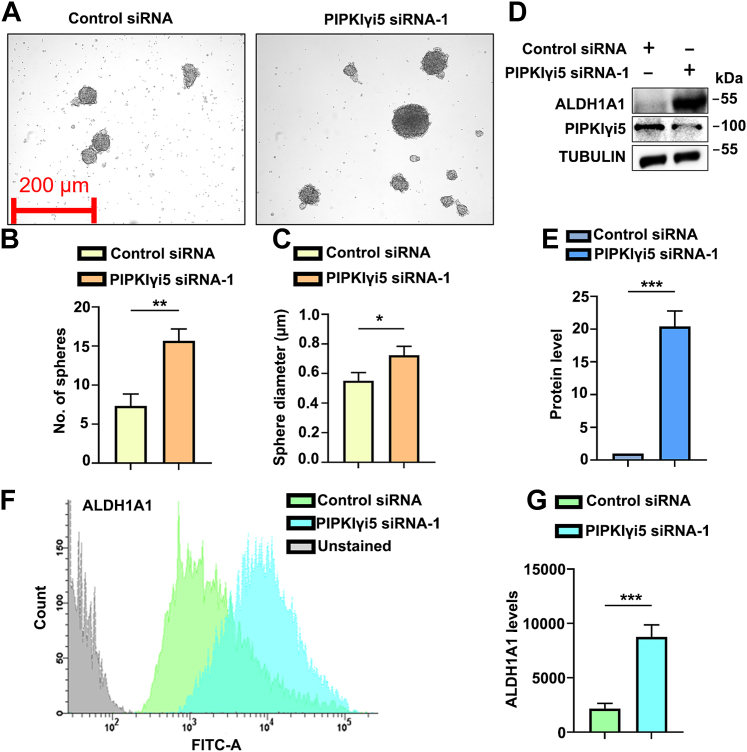
**Loss of PIPKIγi5 promotes the tumorsphere formation.***A*, representative images of sphere formation in control or PIPKIγi5-knockdown CAL27 cells. *B*, quantification of the mean number of spheres generated from 1000 cells plated for sphere formation assay in CAL27 cells. *C*, quantification of the sphere diameter. ALDH1A1 protein levels in spheres derived from control or PIPKIγi5-knockdown CAL27 cells were monitored by Western blot (*D*) and quantified (*E*). ALDH1A1 expression in spheres formed from control or PIPKIγi5-knockdown CAL27 cells was further examined by flow cytometry (*F*) and quantified (*G*). The values shown on graphs represent the mean ± SD from three independent experiments. Unpaired two-tailed Student’s *t* test (*B*, *C*, *E*, and *G*) (∗*p* < 0.05; ∗∗*p* < 0.001; and ∗∗∗*p* < 0.0005). ALDH1A1, Aldehyde Dehydrogenase 1 Family Member A1; PIPKIγi5, type I gamma phosphatidylinositol phosphate kinase i5.

Tumorspheres are spheroid-like structures formed *in vitro* when cancer cells are cultured in a low-attachment environment and in the absence of serum (40). The tumorsphere culture can enrich the subpopulation of cancer cells with stem-like properties, which can grow in anchorage-independent, serum-free environment. YAP1 signaling plays a pivotal role in promoting tumorsphere formation and enhancing cancer stem cell–like properties across various cancers (13, 41, 42). Given that PIPKIγi5 depletion promotes YAP1 signaling, its effects on tumorsphere formation were further investigated. CAL27 cells were transfected with either control or PIPKIγi5-specific siRNA, and a sphere formation assay was conducted. As shown in Figure 8, *A*–*C*, knockdown of PIPKIγi5 increased both the number and size of spheres formed. These findings suggest that the loss of PIPKIγi5 enhances the sphere-formation ability in CAL27 cells. Aldehyde Dehydrogenase 1 Family Member A1 (ALDH1A1) is a well-recognized marker for cancer cell subpopulations exhibiting high ability for sphere formation (43, 44, 45). The expression of ALDH1A1 in tumorspheres derived from control or PIPKIγi5-knockdown cells was evaluated by Western blot (Fig. 8, *D* and *E*) and flow cytometry (Fig. 8, *F* and *G*). Depletion of PIPKIγi5 significantly increased ALDH1A1 expression, supporting that loss of PIPKIγi5 enhances the enrichment of cancer subpopulations with higher sphere formation ability.

**Figure 9:**
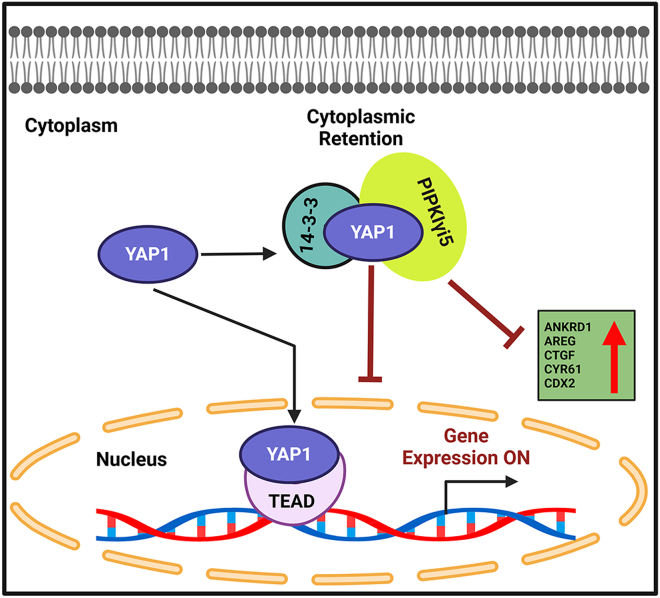
**Model for PIPKIγi5 regulation of Hippo/YAP1 signaling pathway.** PIPKIγi5 interacts with YAP1, inhibiting its nuclear translocation and thereby suppressing YAP1-mediated gene transcription. By facilitating the interaction between YAP1 and 14-3-3 protein, PIPKIγi5 sequesters YAP1 in the cytosol. Therefore, reduction of PIPKIγi5 enhances YAP1 signaling. Schematic diagram was created using BioRender.com. PIPKIγi5, type I gamma phosphatidylinositol phosphate kinase i5; YAP1, Yes-associated protein 1.

Altogether, our data support a model (Fig. 9) in which PIPKIγi5 forms a complex with YAP1 and 14-3-3. Through the production of PI4,5P_2_, PIPKIγi5 facilitates the binding of YAP1 to 14-3-3, thereby sequestering YAP1 in the cytoplasm and preventing its nuclear translocation. Consequently, PIPKIγi5 suppresses YAP1-mediated gene transcription. Depletion of PIPKIγi5 enhances YAP1 nuclear translocation and upregulates YAP1 target gene expression, thereby promoting YAP1-related biological functions.

## Discussion

The Hippo/YAP pathway is highly conserved across species and plays a critical role in regulating cell proliferation, tissue homeostasis, and organ size control (46). Dysregulation of this pathway, such as the overactivation of YAP1, is implicated in various diseases, particularly cancer (47). Consequently, targeting Hippo/YAP signaling has become a prominent focus in the development of novel anticancer therapies, with several drugs showing promise in preclinical studies. Current strategies for developing YAP1-targeting drugs primarily aim to disrupt the YAP1–TEAD interaction or enhance LATS1/2-mediated YAP1 phosphorylation and degradation (16). However, the clinical success of these therapies has been limited, potentially because of an incomplete understanding of the regulators of this pathway.

Our research identifies PIPKIγi5 as a suppressor of YAP1 signaling, promoting the interaction between YAP1 and 14-3-3 to sequester YAP1 in the cytosol. It is well established that the interaction of YAP1 with 14-3-3 is regulated by YAP1 phosphorylation, with LATS1/2-induced phosphorylation at YAP1 S127 being critical for creating a binding site for 14-3-3 proteins (37). Interestingly, PIPKIγi5 does not influence LATS1/2 expression or YAP1 S127 phosphorylation (Fig. 4), suggesting that PIPKIγi5 regulates YAP1 independently of LATS1/2-mediated S127 phosphorylation. Co-IP experiments reveal that 14-3-3 can interact with PIPKIγi5, and this interaction is further enhanced by YAP1 expression (Fig. 5, *C* and *D*). These findings indicate that PIPKIγi5, YAP1, and 14-3-3 can form a complex to regulate YAP1 activation and subcellular translocation.

The specific C-terminal tail of PIPKIγi5 is required for its interaction with YAP1 (Fig. 1). PIPKIγi5 arises *via* alternative splicing of the PIP5K1C gene: all the PIPKIγ variants share the same N-terminal region and kinase domain, but each carries a unique C-terminal extension (18). These tail sequences dictate specific effector binding, subcellular targeting, and distinct biological functions (18). Indeed, only PIPKIγi5, but not PIPKIγi1 or PIPKIγi2, modulates YAP1 signaling (Figs. 7, *C*, *D* and S4), highlighting the critical role of its C-terminal tail. The mechanisms that govern PIP5K1C alternative splicing remain poorly understood. It is worthy to examine how splicing of PIPKIγ is altered in diseases and how those variants affect YAP1 activity for future work.

YAP1 is a PI4,5P_2_ effector protein, with residues R87, K90, and K97 responsible for its binding to PI4,5P_2_ (20). PI4,5P_2_ plays multifaceted roles in regulating YAP1 signaling. In the nucleus, PI4,5P_2_ binding enhances the interaction of YAP1 with the transcription factor TEAD, thereby facilitating target gene expression. Conversely, in the cytosol, PI4,5P_2_ binding promotes the interaction of YAP1 with 14-3-3, inhibiting YAP1 activation. Therefore, the function of PI4,5P_2_ is highly dependent on the subcellular localization of its production, which is mediated by distinct PI4,5P_2_-generating kinases, such as PIPKIα and PIPKIγi5. PIPKIα is critical for mediating nuclear PI4,5P_2_ signaling. By interacting with the WW domain of YAP1 and producing PI4,5P_2_, PIPKIα enhances the YAP1–TEAD interaction (20). Beyond YAP1, PIPKIα also regulates other PI4,5P_2_ effectors, such as p53 and nuclear speckle–targeted PIPKIα-regulated poly(A) polymerase, in the nucleus. PIPKIα associates with p53 to promote its interaction with small heat shock proteins, HSP27 and HSPB5, thereby stabilizing p53 within the nucleus (48). In addition, PIPKIα binds to the nuclear speckle–targeted PIPKIα-regulated poly(A) polymerase to control the expression of select mRNAs, including heme oxygenase-1 (23). In contrast, PIPKIγi5 primarily interacts with its effector proteins in the cytosol. For instance, PIPKIγi5 interacts with small GTPase Rab7a at late endosomes to regulate endosomal trafficking events (32). Similar to its role in sequestering YAP1 in the cytosol, PIPKIγi5 also binds to the interferon (IFN) downstream effector signal transducer and activator of transcription 1 (STAT1), retaining STAT1 in the cytosol (34). This prevents the nuclear translocation of STAT1, thereby blocking IFN-responsive gene transcription.

PIPKIγi5 functions as a suppressor of both YAP1 signaling and IFN signaling, suggesting that these two pathways share common regulatory mechanisms. The interplay between YAP1 signaling and IFN signaling is critical for immune regulation. For instance, IFN-gamma can induce the nuclear translocation and phase separation of YAP1 in cancer cells, thereby diminishing the tumor-immune response and contributing to immunotherapy resistance (49). In addition, lung viral infections elicit a robust IFN-gamma response, which activates the focal adhesion kinase/YAP1 pathway to promote dysplastic cell formation, an essential step in alveolar remodeling in patients with severe viral pneumonia (50). Conversely, YAP1 can also regulate IFN signaling. For example, YAP1 inhibits the production of type I IFN by interacting with IRF3, blocking its dimerization and nuclear translocation, thereby preventing IRF3-mediated type I IFN expression in response to viral infection (51). As PIPKIγi5 regulates both YAP1 signaling and IFN signaling, it provides a critical link between these two pathways. This dual regulatory role highlights PIPKIγi5 as a potentially important modulator of immune responses.

## Experimental procedures

### Cell culture and transfection

UM-SCC-1 cells were from MilliporeSigma (catalog no.: SCC070). CAL27 cells and HEK-293 cells were from American Type Culture Collection. These cells were cultured in Dulbecco's modified Eagle's medium (DMEM) supplemented with 10% fetal bovine serum (FBS). All cell lines were tested for mycoplasma contamination (Beyotime) and used within 6 months. Plasmid transfections were performed using Lipofectamine 3000 (Invitrogen) following the manufacturer’s protocol. For siRNA transfections, cells were transfected using Oligofectamine (Invitrogen) according to the manufacturer’s instructions.

### Reagents

YAP1 human recombinant protein (catalog no.: TP325864) was purchased from OriGene. GST-14-3-3 (catalog no.: 10838-H09E) was from Sino Biological. Antibodies to 14-3-3 HRP (catalog no.: sc-1657HRP) and α-Tubulin (catalog no.: sc398103) were from Santa Cruz Biotechnology. Antibodies to YAP1 (catalog no.: 14074S), GST-tag (catalog no.: 2622S), HA-tag (catalog no.: 3724S), MYC-tag (catalog no.: 2276S), CTGF (catalog no.: 86641T), CYR61 (catalog no.: 14479T), LATS1 (catalog no.: 3477S), LATS2 (catalog no.:5888S), GAPDH (catalog no.: 5174S), phosphorylated YAP1 (Ser127, catalog no.: 13008T), and phosphorylated YAP1 (Ser397, catalog no.: 13619T), Lamin B1 (catalog no.: 12586S), MST1 (catalog no.: 14946), MST2 (catalog no.: 3952), and phosphorylated MST1/2 (catalog no.: 49332) were from Cell Signaling Technology. ALDH1A1 (catalog no.: MA5-29023) antibody was from Invitrogen. Anti-FLAG-Peroxidase antibody (catalog no.: F1804-200UG) was from Sigma–Aldrich. Anti-PIPKIγi2- and PIPKIγi5-specific antibodies were generated as described previously (34). Secondary antibodies were obtained from Jackson ImmunoResearch Laboratories. PI4,5P_2_ diC8 (catalog no.: P-4508) and PI3,5P_2_ diC8 (catalog no.: P-3508) were from Echelon Biosciences. XMU-MP-1 (catalog no.: S8334) was from Selleckchem.

### siRNA

The sequence of control scrambled siRNA was 5′-GUACCUGUACUUCAUGCAG-3′. The sequence of PIPKIγi5 siRNA-1 was 5′-GGAUGGGAGGUACUGGAUU-3′. The sequence of PIPKIγi5 siRNA-2 was 5′-CAGAAGGGCUUUGGGUAA-3′. The siRNA sequence–specific targeting PIPKIγi2 was 5′-GAGCGACACAUAAUUUCUA-3′.

### Recombinant protein purification

HEK-293 cells were transfected with the pHTN Halo tag-HA-PIPKIγi5 plasmid, harvested, and sonicated in buffer (50 mm Tris–HCl, 150 mm NaCl, 1% Nonidet P-40, and 1 mm DTT). The Halo-HA-tagged PIPKIγi5 protein was purified using HaloLink resin and cleaved using HaloTEV Protease.

### *In vitro* binding assay

FLAG-YAP1 (OriGene) (1 μg) and HA-PIPKIγi5 proteins (1 μg) were mixed with assay buffer (20 mM Tris–HCl [pH 7.5], 0.3M NaCl, and 1 mM DTT). The mixture was incubated at 4 °C for 2 h, then anti-FLAG magnetic beads were added into the FLAG-YAP1/HA-PIPKIγi5 mixture, and incubated at 4 °C for 6 h. The beads were washed three times with the assay buffer. The bound proteins were eluted with Laemmli sample buffer and then subjected to Western blot.

### Immunoprecipitation

IP was conducted as previously described (52). Briefly, the IP process involved harvesting and lysing cells in 25 mM Hepes (pH 7.2), 150 mM NaCl, 0.25% NP-40, 1 mM MgCl_2_, and protease inhibitor cocktail. The cells were then incubated with protein G-Sepharose and 2 μg of antibody for 4 h at 4 °C, and the immunocomplexes were separated using SDS-PAGE and analyzed as specified.

### Western blot analysis

Lysates were prepared from cell lines using Radioimmunoprecipitation assay lysis buffer (50 mM Tris–HCl [pH 7.4], 150 mM NaCl, 1% NP-40, 0.25% sodium deoxycholate, 1 mM MEDTA, 1 mM PMSF, 1 mM Na_3_VO_3_, and 1 mM EGTA), and 40 mg of protein lysates were separated using SDS-PAGE. Gels were electroblotted onto polyvinylidene difluoride membranes, blocked with 5% nonfat milk in 1 PBS and 0.1% Tween-20, and probed overnight with primary antibody. Membranes were washed twice and incubated with secondary antibody for 1 h. Enhanced chemiluminescence was used to view the blots with the Syngene G-box (Imgen Technologies), and quantification was performed using ImageJ (National Institutes of Health) software with a minimum of three independent experiments.

### RNA extraction and real-time PCR

Total RNA was extracted using the Isolate II RNA Mini Kit (Bioline) in accordance with the manufacturer's guidelines. Subsequently, complementary DNA synthesis was conducted using a complementary DNA Reverse Transcription Kit (ThermoFisher Scientific). Endogenous gene mRNA levels were quantified using SYBR Green (Power-UP SYBR-Green PCR Master Mix; Applied Biosystems) and evaluated using the Quantstudio3 Real-time PCR system (Applied Biosystems). Expression data were standardized to control GAPDH, and mRNA fold changes were computed using the 2^-ΔΔCT^ method. Primers used for the PCR were as follows: 5′-GCACCTGGAAGCAGTAACATGC-3′ (forward) and 5′-GGCAGCTATGGCTGCTAATGCA-3′ (reverse) for AREG; 5′-CCAATGACAACGCCTCCTG-3′ (forward) and 5′-TGGTGCAGCCAGAAAGCTC-3′ (reverse) for CDX2; 5′-AAAAGTGCATCCGTACTCCCA-3′ (forward) and 5′-CCGTCGGTACATACTCCACAG-3′ (reverse) for CTGF; 5′-CGACTCCTGATTATGTATGGCGC-3′ (forward) and 5′-GCTTTGGTTCCATTCTGCCAGTG-3′ (reverse) for ANKRD1; 5′-AGCCTCGCATCCTATACAACC-3′ (forward) and 5′-TTCTTTCACAAGGCGGCACTC-3′ (reverse) for CYR61; and 5′-GTCTCCTCTGACTTCAACAGCG-3′ (forward) and 5′-ACCACCCTGTTGCTGTAGCCAA-3′ (reverse) for GAPDH.

### Cytoplasmic and nuclear extract preparation

The Nuclear/Cytosol Fractionation Kit (Abcam) was used to prepare cytoplasmic and nuclear extracts in accordance with the manufacturer’s guidelines.

### Immunofluorescence

Cells were resuspended and plated on coverslips in DMEM supplemented with 10% FBS and allowing for adhesion over a period of 12 h. The cells were rinsed with PBS and then fixed with chilled methanol at −20 °C. Then, the cells were rinsed with PBS and permeabilized with 0.5% Triton X-100 and subsequently blocked with 3% bovine serum albumin (BSA) in PBS at room temperature for 60 min, followed by overnight incubation with the primary antibody at 4 °C. The cells were rinsed with 0.1% Triton X-100 in PBS and incubated with a fluorescence-conjugated secondary antibody at room temperature for 60 min. The cell nuclei were stained with 4′,6-diamidino-2-phenylindole solution for 5 min, and then they were washed with 0.1% Triton X-100 in PBS. Coverslips were affixed with VECTASHIELD mounting media and analyzed using a 100× oil immersion objective on a laser scanning confocal microscope (LSM 710; Carl Zeiss). Images were analyzed and quantified utilizing ImageJ software.

### Solid-phase binding assay

This experiment was conducted as previously described by Ghosh *et al.* (34). GST-14-3-3 or HA-PIPKIγi5 (1 μg) was coated onto Microtiter plates (96 wells; MaxiSorp Immuno Plate, Nunc) for overnight at 4 °C and then blocked for 1 h at rom temperature using 1% fatty acid–free BSA in PBS. The plates were then incubated with or without PI4,5P_2_ or PI4P for 30 min at room temperature, followed by incubation for 1 h at room temperature with 1 μg of FLAG-YAP1. The wells were washed three times with PBS containing 1% fatty acid–free BSA, bound proteins were eluted with Laemmli sample buffer, and the plate was incubated at 95 °C for 7 min.

### Sphere formation assay

Cells were cultured (2 × 10^4^/well) in serum-free DMEM (Life Technologies) supplemented with B-27 (Life Technologies), 20 ng/ml basic fibroblast growth factor (ThermoFisher), and 20 ng/ml epidermal growth factor (ThermoFisher) to induce sphere formation. Cell culture was conducted in ultralow attachment 6-well plates (Corning) for 10 days, and sphere formation was evaluated using an inverted phase-contrast microscope (Axio Observer; Carl Zeiss) at 20× magnification. A single sphere larger than 100 μm (diameter) was counted, images of spheres were captured, and analysis was carried out with ImageJ software.

### Flow cytometry

Flow cytometric analysis was carried out to determine the percentage of ALDH1A1-positive cells in the spheres generated from control or PIPKIγi5-knockdown CAL27 cell lines. Cells were harvested and resuspended in dilution buffer (PBS, 2% FBS and 0.1% BSA) and then permeabilized with 0.1% Triton X-100. Then, ALDH1A1 primary antibody was added to each sample and incubated for 2 h at 4 °C. After incubation, cells were washed three times with ice-cold PBS and incubated with a fluorescence-conjugated secondary antibody for 30 min at room temperature. Then cells were washed three times with ice-cold PBS and resuspended in dilution buffer. Flow cytometric analysis was performed using BD FACSymphony A1 (BD Biosciences), and analysis was carried out with BD FACSDiva software.

### Statistical analysis

Data analysis was conducted utilizing Prism 10 (GraphPad) for Windows. Bar graphs illustrate the mean along with the standard deviation, as specified. The unpaired two-tailed Student’s *t* test and one-way ANOVA with multiple comparisons were employed to evaluate statistical significance. Statistical significance was assessed with ∗*p* < 0.05; ∗∗*p* < 0.001; ∗∗∗*p* < 0.0005; and ∗∗∗∗*p* < 0.0001. Figures indicate nonsignificant differences with the notation ns.

## Data availability

All the data generated or analyzed during the study are included in the article and its associated supporting information.

## Supporting information

This article contains supporting information.

## Conflict of interest

The authors declare that they have no conflicts of interest with the contents of this article.
