# Supplementary material for: Type I gamma phosphatidylinositol phosphate kinase i5 suppresses YAP1 signaling
Source: J Biol Chem. 2025 Aug 8;301(9):110573. doi: 10.1016/j.jbc.2025.110573 (PMC12444467; doi:10.1016/j.jbc.2025.110573)
Supplement: Supporting Figures [file mmc1.pdf]

Supporting information for:

Type I gamma phosphatidylinositol phosphate kinase i5 suppresses YAP1 signaling

Chinmoy Ghosh[1], Ruchi Kakar[1], Matthew Bavuso[1], Huizhi Wang[1,2], and Yue Sun[1,2,†]

1. Department of Oral and Craniofacial Molecular Biology, Philips Institute for Oral Health Research, School of Dentistry, Virginia Commonwealth University, Richmond, VA 23298, USA

2. Massey Cancer Center, Virginia Commonwealth University, Richmond, VA 23298, USA

[†]To whom correspondence should be addressed. E-mail: ysun4@vcu.edu

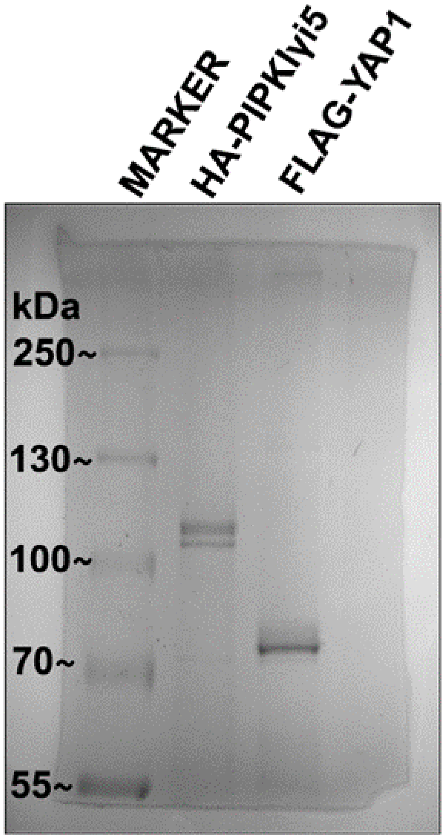

Figure S1. Coomassie Blue staining of purified YAP1 and PIPKIγi5. Purified FLAG-YAP1 (1 µg) and HA-PIPKIγi5 (1 µg) recombinant proteins were separated by SDS-PAGE gel and stained with Coomassie Blue.

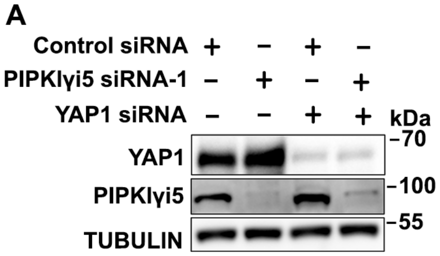

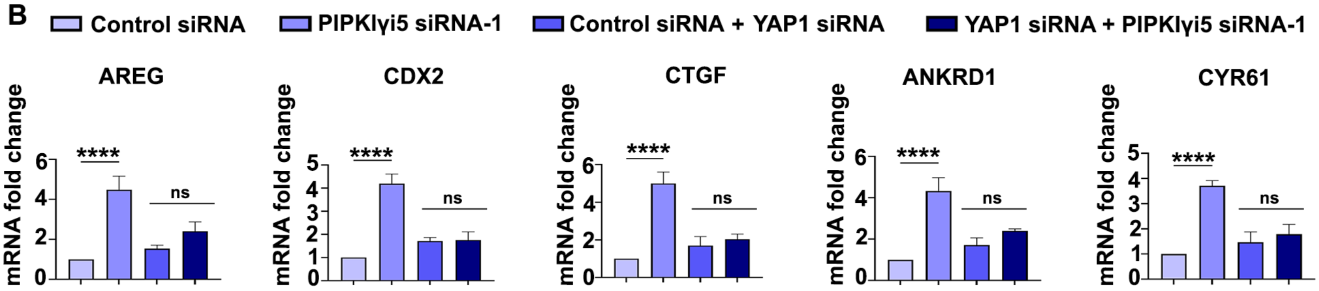

Figure S2. Effects of PIPKIγi5 on gene expression is dependent on YAP1. Control siRNA or PIPKIγi5 siRNA-1 were co-transfected with or without YAP1 siRNA into CAL27 cells. (A) YAP1 and PIPKIγi5 protein levels were examined by Western blot. (B) The mRNA levels of indicated YAP1 target genes were examined by Real-time PCR. The values shown on graphs represent the mean ± SD from three independent experiments. One-way ANOVA and Tukey's HSD (B) (****$p < 0.0001$; and ns, non-significant).

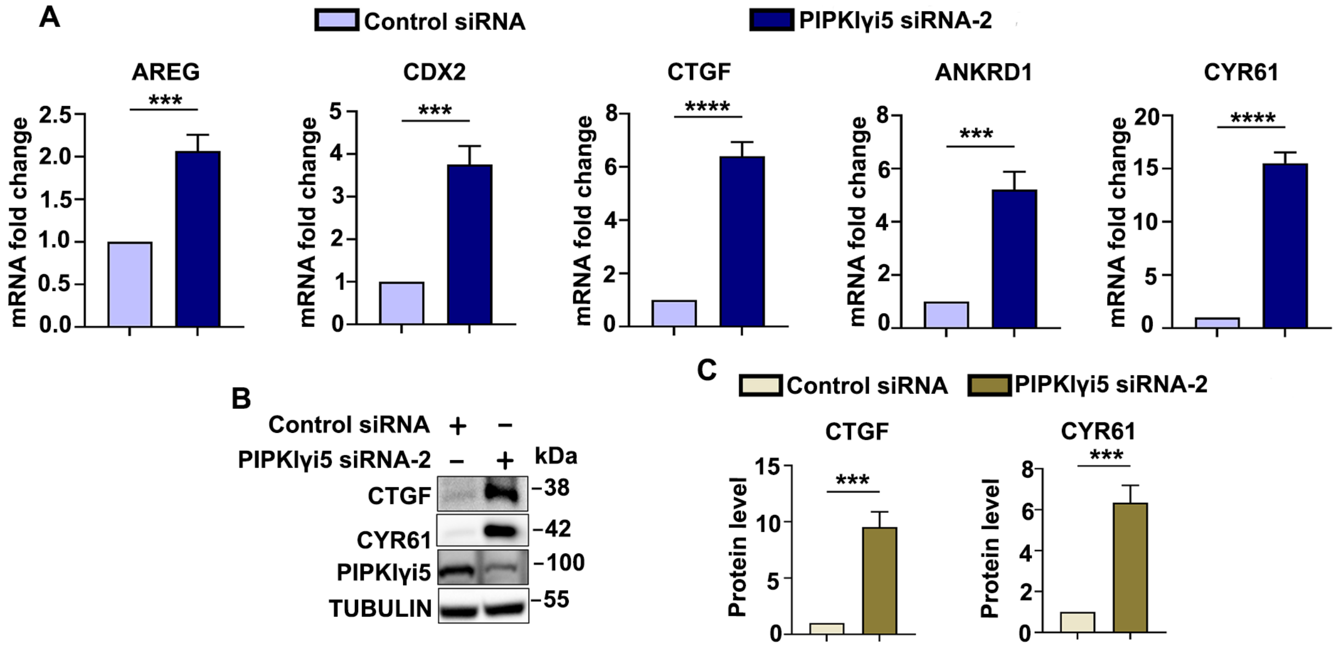

Figure S3. Effects of PIPKIγi5 siRNA-2 on YAP1 target gene expression. Control siRNA or PIPKIγi5 siRNA-2 was transfected into CAL27 cells. (A) The mRNA levels of indicated YAP1 target genes were examined by Real-time PCR. (B) CTGF, CYR61, and PIPKIγi5 protein levels were examined by Western blot. (C) Protein levels of CTGF and CYR61 were normalized with Tubulin levels and quantified. The values shown on graphs represent the mean ± SD from three independent experiments. Unpaired two-tailed Student's *t*-test (A, C) (***$p < 0.0005$; ****$p < 0.0001$).

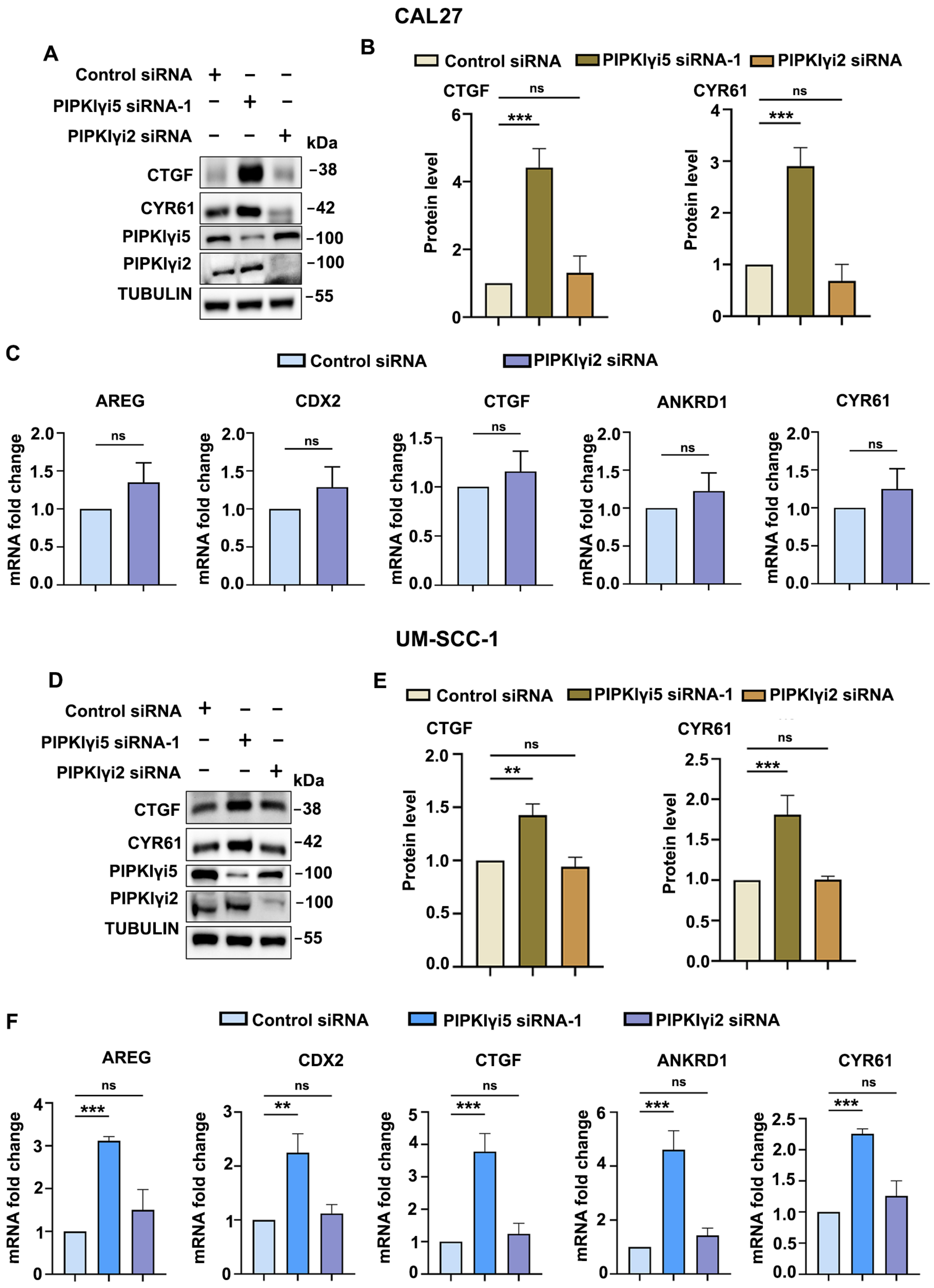
CAL27
A
Control siRNA
PIPKIγi5 siRNA-1
PIPKIγi2 siRNA
kDa
CTGF
CYR61
PIPKIγi5
PIPKIγi2
TUBULIN
B
Control siRNA
PIPKIγi5 siRNA-1
PIPKIγi2 siRNA
CTGF
CYR61
Protein level
C
Control siRNA
PIPKIγi2 siRNA
AREG
CDX2
CTGF
ANKRD1
CYR61
mRNA fold change
UM-SCC-1
D
Control siRNA
PIPKIγi5 siRNA-1
PIPKIγi2 siRNA
kDa
CTGF
CYR61
PIPKIγi5
PIPKIγi2
TUBULIN
E
Control siRNA
PIPKIγi5 siRNA-1
PIPKIγi2 siRNA
CTGF
CYR61
Protein level
F
Control siRNA
PIPKIγi5 siRNA-1
PIPKIγi2 siRNA
AREG
CDX2
CTGF
ANKRD1
CYR61
mRNA fold change

Figure S4. Loss of PIPKIγi5 enhances YAP1 target genes expression in CAL27 and UM-SCC-1 cells. Control siRNA, PIPKIγi5 siRNA-1, or PIPKIγi2 siRNA were transfected into CAL27 cells. (A) The levels of protein expression of the YAP1 target genes CTGF and CYR61 in control or PIPKIγi5-knockdown CAL27 cells were monitored by Western blot. (B) Protein levels of CTGF and CYR61 were normalized with Tubulin levels and quantified. (C) The mRNA levels of indicated YAP1 target genes in control or PIPKIγi2-knockdown CAL27 cells were examined by Real-time PCR. Control siRNA, PIPKIγi5 siRNA-1, or PIPKIγi2 siRNA were transfected into UM-SCC-1 cells, and then the levels of protein expression of the YAP1 target genes CTGF and CYR61 in these UM-SCC-1 cells were monitored by Western blot (D) and quantified (E). The mRNA levels of indicated YAP1 target genes in UM-SCC-1 cells were examined by Real-time PCR (F). The values shown on graphs represent the mean ± SD from three independent experiments. One-way ANOVA and Tukey's HSD (B, E, F) (** $p < 0.001$; *** $p < 0.0005$; and ns, non-significant). Unpaired two-tailed Student's *t*-test (C) (ns, non-significant).

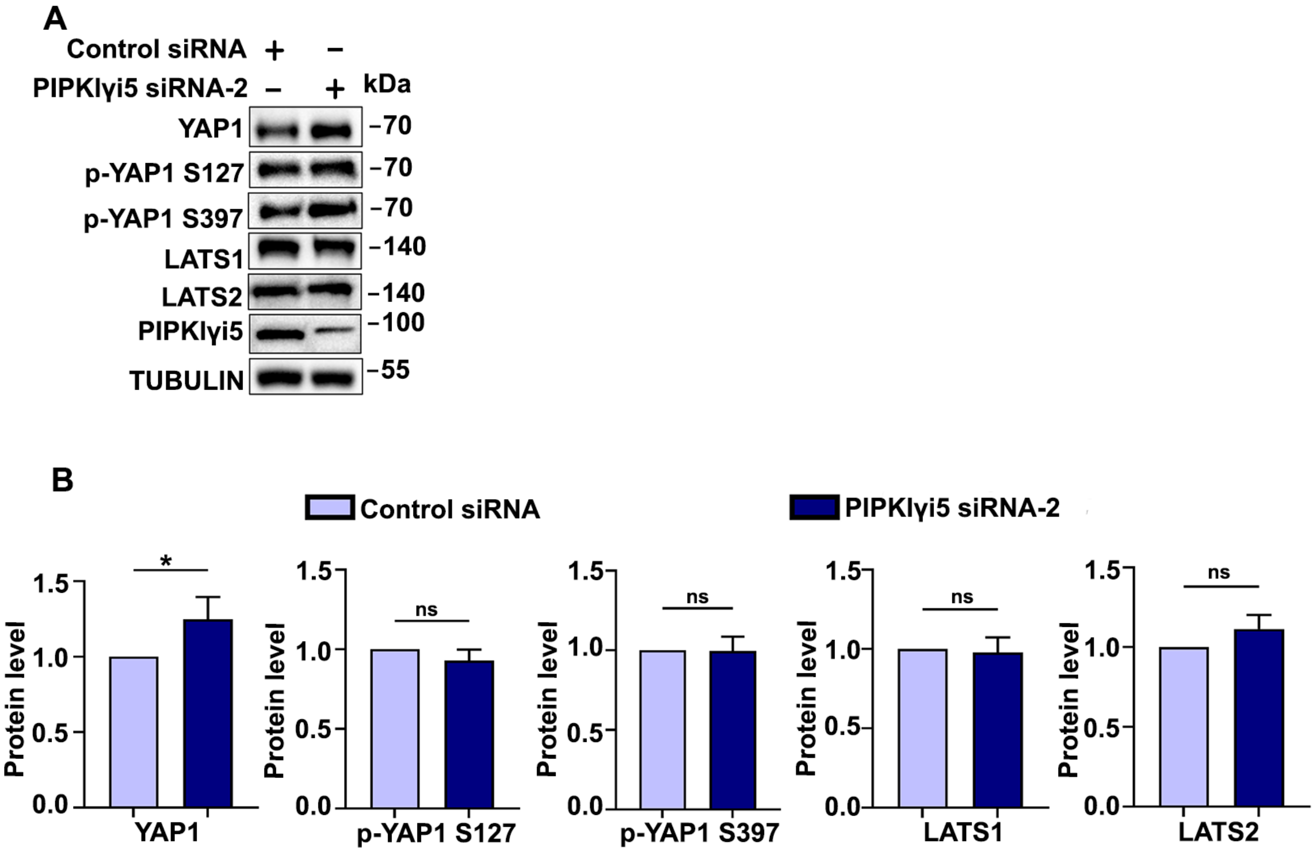

Figure S5. Effects of PIPKIγi5 siRNA-2 on YAP1 expression and phosphorylation. CAL27 cells were transfected with control siRNA or PIPKIγi5 siRNA-2, and the specified protein levels were analyzed using Western blot (A). Quantification of total YAP1, YAP1 phosphorylation (S127 and S397), LATS1 and LATS2 in CAL27 cells (B). The values shown on graphs represent the mean ± SD from three independent experiments. Unpaired two-tailed Student's *t*-test (B) (*$p < 0.05$; and ns, non-significant).

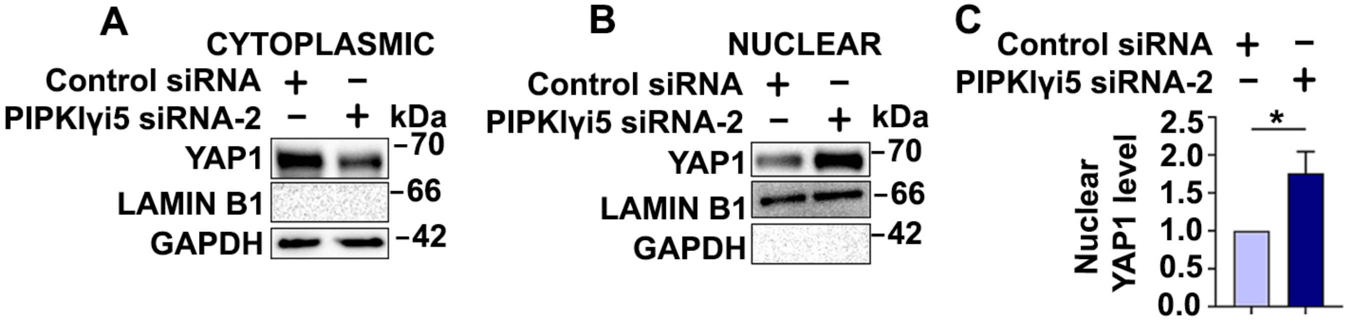

Figure S6. Effects of PIPKIγi5 siRNA-2 on YAP1 nuclear translocation. CAL27 cells were transfected with either control siRNA or PIPKIγi5 siRNA-2, and the cytoplasmic and nuclear fractions were isolated using nuclear extraction kits. (A) Cytoplasmic components were subjected to immunoblotting using indicated antibodies. (B) Nuclear components were subjected to immunoblotting using specified antibodies. (C) Quantification of the levels of nuclear YAP1 in control or PIPKIγi5-knockdown CAL27 cells. Nuclear YAP1 levels were normalized with Lamin B1 levels. The values shown on graphs represent the mean ± SD from three independent experiments. Unpaired two-tailed Student's *t*-test (C) (*$p < 0.05$).
